# Evaluation of Placental and Fetal Tissue Specimens for Zika Virus Infection — 50 States and District of Columbia, January–December, 2016

**DOI:** 10.15585/mmwr.mm6624a3

**Published:** 2017-06-23

**Authors:** Sarah Reagan-Steiner, Regina Simeone, Elizabeth Simon, Julu Bhatnagar, Titilope Oduyebo, Rebecca Free, Amy M. Denison, Demi B. Rabeneck, Sascha Ellington, Emily Petersen, Joy Gary, Gillian Hale, M. Kelly Keating, Roosecelis B. Martines, Atis Muehlenbachs, Jana Ritter, Ellen Lee, Alexander Davidson, Erin Conners, Sarah Scotland, Kayleigh Sandhu, Andrea Bingham, Elizabeth Kassens, Lou Smith, Kirsten St. George, Nina Ahmad, Mary Tanner, Suzanne Beavers, Brooke Miers, Kelley VanMaldeghem, Sumaiya Khan, Ingrid Rabe, Carolyn Gould, Dana Meaney-Delman, Margaret A. Honein, Wun-Ju Shieh, Denise J. Jamieson, Marc Fischer, Sherif R. Zaki, U.S. Zika Pregnancy Registry Collaboration, Zika Virus Response Epidemiology and Surveillance Task Force Pathology Team, U.S. Zika Pregnancy Registry Collaboration, Melissa Kretschmer, Kara Tarter, Hayley Yaglom, Shoruq Alhajmohammad, Dildeep Chhabra, Wendy Jilek, Meghana Madala, Sharon Messenger, Charsey Cole Porse, Maria Salas, Diana Singh, Sarah Skallet, Similoluwa Sowunmi, Natalie S. Marzec, Karin Davis, Brenda Esponda-Morrison, M. Zachariah Fraser, Colleen Ann O'Connor, Wendy M. Chung, Folasuyi Richardson, Meredith E. Stocks, Amanda Marie Bundek, Jennifer L. Zambri, Ashley Allen, Marie Ketty Etienne, Jennifer Jackson, Vanessa Landis, Teresa Logue, Nicole Muse, Juliana Prieto, Mercedes Rojas, Amanda Feldpausch, Teri Graham, Sylvia Mann, Sarah Y. Park, Debbie Freeman, Emily J. Potts, Taryn Stevens, Sean Simonson, Julius L. Tonzel, Shari Davis, Sara Robinson, Judie K. Hyun, Erin Maureen Jenkins, Catherine Brown, Susan Soliva, Elizabeth Schiffman, Paul Byers, Sheryl Hand, Christine L. Mulgrew, Jeff Hamik, Samir Koirala, Elizabeth Ludwig, Carolyn R. Fredette, Abigail A. Mathewson, Kristin Garafalo, Karen Worthington, Abubakar Ropri, Danielle Bloch, Sandhya Clark, Hannah Cooper, Annie D. Fine, Gili Hrusa, Martha Iwamoto, Hannah Kubinson, Christopher T. Lee, Sally Slavinski, Eliza Wilson, Ann Winters, David Yi Yang, Julius N. Ade, Zahra Alaali, Kimberly Alvarez, P. Bryon Backenson, Debra Blog, Amy Dean, Elizabeth Dufort, Andrea Marias Furuya, Meghan Fuschino, Rene Hull, Matthew Kleabonas, Karen Kulas, Philip Kurpiel, Lou Ann Lance, Emaly Leak, Ronald J. Limberger, Stephanie Ostrowski, MaryJo Polfleit, Amy Robbins, Jemma V. Rowlands, Inderbir Sohi, Jamie N. Sommer, Jennifer White, Dorothy Wiley, Li Zeng, Ronna L. Chan, Jennifer MacFarquhar, Laura Cronquist, Leah Lind, Kumar Nalluswami, Dana Perella, Diane S. Brady, Michael Gosciminski, Patricia McAuley, Bridget E. Teevan, Daniel Drociuk, Vinita Leedom, Brian Witrick, Jan Bollock, Lon Kightlinger, Marie Bottomley Hartel, Loraine Swanson Lucinski, Morgan McDonald, Angela M. Miller, Tori Armand Ponson, Laura Price, Kelly Broussard, Amy E. Nance, Dallin Peterson, Brennan Martin, Shea Browne, LaToya A. Griffin-Thomas, Jennifer O. Macdonald, Jillian Neary, Hanna Oltean, Alys Adamski, Madelyn Baez-Santiago, Brigid C. Bollweg, Janet D. Cragan, Yokabed Ermias, Lindsey B. C. Estetter, Shannon Fleck-Derderian, Cynthia S. Goldsmith, Matthew R. Groenewold, Heather Hayes, Irogue Igbinosa, Tiffany Gayle Jenkinson, Abbey M. Jones, Amanda Lewis, Cynthia A. Moore, Kimberly B. Newsome, Vaunita Parihar, Mitesh M. Patel, Anna Paulino, Sonja A. Rasmussen, Meghan Raycraft, Megan R Reynolds, Dominique C. Rollin, Jeanine H. Sanders, Carrie Shapiro-Mendoza, Luciana Silva-Flannery, Pamela Spivey, Alphonse K. Tshiwala, Tonya R. Williams, Zika Virus Response Epidemiology and Surveillance Task Force Pathology Team, William A. Bower, Elizabeth Davlantes, Terra R. Forward, Rena Fukunaga, Jonas Hines, Shaohua Sean Hu, Jessica Leung, Lillianne Lewis, Stacey Martin, Lucy McNamara, John D. Omura, Candice L. Robinson, Kristine Schmit, Julie L. Self, Minesh Shah, Anne Straily, Elizabeth A. Van Dyne, Milan Vu, Charnetta Williams

**Affiliations:** ^1^Division of High-Consequence Pathogens and Pathology, National Center for Emerging and Zoonotic Infectious Diseases, CDC; ^2^Division of Congenital and Developmental Disorders, National Center on Birth Defects and Developmental Disabilities, CDC; ^3^Division of Reproductive Health, National Center for Chronic Disease Prevention and Health Promotion, CDC; ^4^Division of Emergency Operations, Office of Public Health Preparedness and Response, CDC; ^5^New York City Department of Health & Mental Hygiene; ^6^Massachusetts Department of Public Health; ^7^Florida Department of Health, ^8^New York State Department of Health; ^9^Epidemic Intelligence Service, CDC; ^10^Division of HIV/AIDS Prevention, National Center for HIV/AIDS, Viral Hepatitis, STD, and TB Prevention, CDC; ^11^Division of Environmental Hazards and Health Effects, National Center for Environmental Health, CDC; ^12^Oak Ridge Institute for Science and Education; ^13^Division of Vector-Borne Infectious Diseases, National Center for Emerging and Zoonotic Infectious Diseases, CDC; ^14^Office of the Director, National Center for Emerging and Zoonotic Infectious Diseases, CDC.; Maricopa County Department of Public Health; Arizona Department of Health Services; Arizona Department of Health Services; Arizona Department of Health Services; California Department of Public Health; California Department of Public Health; California Department of Public Health; California Department of Public Health; California Department of Public Health; California Department of Public Health; California Department of Public Health; California Department of Public Health; California Department of Public Health; California Department of Public Health; Colorado Department of Public Health and Environment; Connecticut Department of Public Health; Connecticut Department of Public Health; Connecticut Department of Public Health; Connecticut Department of Public Health; Dallas County Health and Human Services; Dallas County Health and Human Services; Dallas County Health and Human Services; Delaware Division of Public Health; Delaware Division of Public Health; Florida Department of Health, Bureau of Public Health Laboratories-Miami; Florida Department of Health in Miami-Dade County; Florida Department of Health in Orange County; Florida Department of Health; Florida Department of Health in Miami-Dade County; Florida Department of Health in Miami-Dade County; Florida Department of Health; Florida Department of Health in Miami-Dade County; Georgia Department of Public Health; Georgia Department of Public Health; Hawaii Department of Health; Hawaii Department of Health; Illinois Department of Public Health; Indiana State Department of Health; Indiana State Department of Health; Louisiana Department of Health; Louisiana Department of Health; Maine Department of Health and Human Services; Maine Department of Health and Human Services; Maryland Department of Health and Mental Hygiene; Maryland Department of Health and Mental Hygiene; Massachusetts Department of Public Health; Massachusetts Department of Public Health; Minnesota Department of Health; Mississippi State Department of Health; Mississippi State Department of Health; Montana Department of Health and Human Services; Division of Public Health; Nebraska Department of Health and Human Services; Division of Public Health; Nebraska Department of Health and Human Services; Division of Public Health; Nebraska Department of Health and Human Services; New Hampshire Department of Health and Human Services; New Hampshire Department of Health and Human Services; New Jersey Department of Health; New Jersey Department of Health; New Mexico Department of Health; New York City Department of Health & Mental Hygiene; New York City Department of Health & Mental Hygiene; New York City Department of Health & Mental Hygiene; New York City Department of Health & Mental Hygiene; New York City Department of Health & Mental Hygiene; New York City Department of Health & Mental Hygiene; New York City Department of Health & Mental Hygiene; New York City Department of Health & Mental Hygiene; New York City Department of Health & Mental Hygiene; New York City Department of Health & Mental Hygiene; New York City Department of Health & Mental Hygiene; New York City Department of Health & Mental Hygiene; New York State Department of Health; New York State Department of Health; New York State Department of Health; New York State Department of Health; New York State Department of Health; Wadsworth Center; New York State Department of Health; New York State Department of Health; Wadsworth Center; New York State Department of Health; Wadsworth Center; New York State Department of Health; Wadsworth Center; New York State Department of Health; Wadsworth Center; New York State Department of Health; Wadsworth Center; New York State Department of Health; New York State Department of Health; New York State Department of Health; Wadsworth Center; New York State Department of Health; Wadsworth Center; New York State Department of Health; New York State Department of Health; New York State Department of Health; New York State Department of Health; Bureau of Communicable Disease Control; New York State Department of Health; New York State Department of Health, CDC; New York State Department of Health; Bureau of Communicable Disease Control; New York State Department of Health; New York State Department of Health; Wadsworth Center; New York State Department of Health; North Carolina Department of Health and Human Services; Division of Public Health; North Carolina Department of Health and Human Services; Division of Public Health; North Dakota Department of Health; Pennsylvania Department of Health; Pennsylvania Department of Health; Philadelphia Department of Public Health; Rhode Island Department of Health; Rhode Island Department of Health; Rhode Island Department of Health; Rhode Island Department of Health; South Carolina Department of Health and Environmental Control; South Carolina Department of Health and Environmental Control; South Carolina Department of Health and Environmental Control; South Dakota Department of Health; South Dakota Department of Health; Tennessee Department of Health; Tennessee Department of Health; Tennessee Department of Health; Tennessee Department of Health; Tennessee Department of Health; Tennessee Department of Health; Texas Department of State Health Services; Utah Birth Defect Network; Utah Department of Health; Utah Department of Health; Vermont Department of Health; Virginia Department of Health; Virginia Division of Consolidated Laboratory Services; Virginia Department of Health; Washington State Department of Health; Washington State Department of Health; CDC; CDC; CDC; CDC; CDC; CDC; CDC, ORISE; CDC; CDC; CDC; CDC; CDC; CDC; CDC; CDC; CDC; CDC; CDC; CDC; CDC; CDC; CDC; CDC; CDC; CDC; CDC; CDC; CDC; CDC.; CDC; CDC, Epidemic Intelligence Service (EIS; CDC; CDC, EIS; CDC; CDC; CDC; CDC; CDC; CDC; CDC; CDC; CDC; CDC, EIS; CDC; CDC, EIS; CDC; CDC; CDC, EIS.

Zika virus infection during pregnancy can cause congenital microcephaly and brain abnormalities (*1*), and detection of Zika virus RNA in clinical and tissue specimens can provide definitive laboratory evidence of recent Zika virus infection. Whereas duration of viremia is typically short, prolonged detection of Zika virus RNA in placental, fetal, and neonatal brain tissue has been reported and can provide key diagnostic information by confirming recent Zika virus infection (*2*). In accordance with recent guidance (*3*,*4*), CDC provides Zika virus testing of placental and fetal tissues in clinical situations where this information could add diagnostic value. This report describes the evaluation of formalin-fixed paraffin-embedded (FFPE) tissue specimens tested for Zika virus infection in 2016 and the contribution of this testing to the public health response. Among 546 live births with possible maternal Zika virus exposure, for which placental tissues were submitted by the 50 states and District of Columbia (DC), 60 (11%) were positive by Zika virus reverse transcription–polymerase chain reaction (RT-PCR). Among 81 pregnancy losses for which placental and/or fetal tissues were submitted, 18 (22%) were positive by Zika virus RT-PCR. Zika virus RT-PCR was positive on placental tissues from 38/363 (10%) live births with maternal serologic evidence of recent unspecified flavivirus infection and from 9/86 (10%) with negative maternal Zika virus immunoglobulin M (IgM) where possible maternal exposure occurred >12 weeks before serum collection. These results demonstrate that Zika virus RT-PCR testing of tissue specimens can provide a confirmed diagnosis of recent maternal Zika virus infection.

Zika virus RT-PCR and, in selected cases, immunohistochemical (IHC) testing, were performed at CDC’s Infectious Diseases Pathology Branch (IDPB) on FFPE tissue specimens submitted from completed pregnancies (i.e., live births and pregnancy losses of any gestational age) with possible maternal Zika virus exposure.* Completed pregnancies in this report include those with evidence of possible recent Zika virus infection (from maternal, fetal, or infant specimens) and those that ultimately demonstrated no laboratory evidence of possible Zika virus infection. To determine the added diagnostic value of Zika virus tissue RT-PCR testing, results from nontissue clinical samples (i.e., serum and/or urine) reported by the submitting health department or CDC’s Arboviral Diseases Branch, were categorized by maternal test results (Table 1) (*5*) and infant test results.^†^ Tissue RT-PCR results are also summarized by maternal symptom status and trimester of infection or possible exposure.^§^ A subset of pregnancies that were also reported to the U.S. Zika Pregnancy Registry (USZPR)^¶^ were systematically reviewed to determine the presence of possible Zika virus–associated birth defects. Thus, the analysis of tissue RT-PCR results by the presence of possible birth defects was limited to these pregnancies. Infants and pregnancy losses with possible Zika virus–associated birth defects included pregnancies completed by December 25, 2016 that were reported to the USZPR and met the CDC surveillance case definition for possible Zika virus–associated birth defects as of May 18, 2017.** Completed pregnancies were classified as “tissue Zika virus RT-PCR–positive” if at least one placental (e.g., placental disc, umbilical cord, or fetal membranes) specimen or fetal/infant tissue specimen was positive by conventional Zika virus RT-PCR and confirmed by sequencing of PCR products (*2*). A positive Zika virus RT-PCR test result on placental tissues is evidence of maternal Zika virus infection. This report includes cases reported previously (*2*,*6*–*8*).

**TABLE 1:** Categories for laboratory evidence of maternal Zika virus infection from testing of nontissue clinical samples (e.g., serum, urine)

Category	Definition
Confirmed recent Zika virus infection	Positive Zika virus RT-PCR, or Zika or dengue virus IgM positive or equivocal* with Zika virus PRNT titer ≥10 and dengue virus PRNT titer <10
Recent unspecified flavivirus infection	Zika virus RT-PCR negative or not performed, with Zika or dengue virus IgM positive, or equivocal with Zika virus and dengue virus PRNT titers ≥10
Maternal samples negative by Zika virus IgM, all or part of possible exposure occurred >12 weeks before serum collection	Zika virus RT-PCR negative or not performed, with Zika virus IgM negative, where all or part of possible maternal exposure occurred >12 weeks before serum collection date
Pending/Unknown	Test results unknown or pending
No evidence of Zika virus infection	Zika or dengue IgM positive or equivocal with Zika virus PRNT titer <10 regardless of dengue PRNT titer, or Zika virus IgM negative where all possible exposure occurred within 2–12 weeks of serum collection date
No maternal clinical samples tested	No maternal serum, urine, or other clinical specimens tested

**Abbreviations:** IgM = immunoglobulin M; PRNT = plaque-reduction neutralization test; RT-PCR = reverse transcription–polymerase chain reaction.

* Serology terminology varies by assay and nonnegative results can include positive, equivocal, presumptive positive, or possible positive results.

During 2016, tissue specimens from 627 completed pregnancies with possible maternal Zika virus exposure from the 50 states and DC were submitted to CDC and were tested by Zika virus tissue RT-PCR. These specimens included placental tissues from 546 live births and placental and/or fetal tissues from 81 pregnancy losses; IHC testing for Zika virus was also performed on specimens from 91 live births and pregnancy losses (15%), criteria for which are specified below. Overall, 78/627 (12%) had one or more placental or fetal tissue specimen that was positive for Zika virus by RT-PCR. Among the 91 completed pregnancies with tissue specimens tested by IHC, seven (8%) demonstrated IHC evidence of Zika virus infection (six from first trimester pregnancy losses and one from a second trimester pregnancy loss). All seven IHC-positive pregnancy losses were also tissue RT-PCR–positive. Because none of the placental specimens tested by IHC from third trimester pregnancy losses (n = 4) or live births (n = 47) was IHC-positive, beginning in March 2016, IHC testing of these specimen types was no longer routinely performed.

Among 546 live births, placental tissues from 60 (11%) were RT-PCR positive for Zika virus, including 38/363 (10%) from pregnancies with recent unspecified maternal flavivirus infection and 9/86 (10%) with negative maternal Zika virus IgM, where possible maternal exposure occurred >12 weeks before serum collection (after which time maternal Zika virus IgM antibodies might have waned) (*5*) (Table 2). Zika virus RT-PCR was negative on placental tissues from 34/47 (72%) live births with confirmed recent maternal Zika virus infection, and from all three live births in which the infant had confirmed congenital Zika virus infection based on infant testing. Among live births with no evidence of maternal Zika virus infection (n = 14) or no maternal clinical specimens tested (n = 34), none was tissue RT-PCR–positive. Overall, Zika virus RT-PCR was positive on placental tissues from 47/482 (10%) live births without a confirmed diagnosis by Zika virus testing on maternal or infant clinical specimens, confirming a diagnosis of recent maternal Zika virus infection (Figure).

**TABLE 2:** Zika virus RT-PCR results from fixed placental and fetal tissue samples from completed pregnancies for which specimens* were submitted to CDC’s Infectious Diseases Pathology Branch, by pregnancy outcome — 50 U.S. states and District of Columbia (n = 627), including 449 reported to the U.S. Zika Pregnancy Registry, January–December 2016

All completed pregnancies from which tissue specimens were submitted (n = 627)				
Characteristic	Live births (n = 546)		Pregnancy losses (n = 81)	
	Live births with tissue specimens tested, no.	Tissue RT-PCR positive,^†^ no. (%)	Pregnancy losses with tissue specimens tested, no.	Tissue RT-PCR positive, no. (%)
**Total**	**546**	**60 (11)**	**81**	**18 (22)**
**Maternal clinical Zika virus test results^§^**				
Confirmed recent Zika virus infection	47	13 (28)	19	11 (58)
Recent unspecified flavivirus infection	363	38 (10)	13	4 (31)
Maternal samples negative by Zika virus IgM, all or part of possible exposure occurred >12 weeks before serum collected^¶^	86	9 (10)	18	2 (11)
No maternal clinical samples tested**	34	—	16	1 (6)
Pending/Unknown	2	—	1	—
No evidence of possible Zika virus infection	14	—	14	—
**Infant clinical Zika virus test results** ^††^				
Confirmed congenital Zika virus infection	3	—	NA	NA
Probable congenital Zika virus infection	46	9 (20)	NA	NA
Negative Zika virus testing	358	39 (11)	NA	NA
No results reported	139	12 (9)	NA	NA
**Trimester of infection or possible exposure** ^§§^				
First trimester only	90	9 (10)	41	12 (29)
Multiple trimesters, including first	291	32 (11)	24	4 (17)
Second and/or third trimester only	149	18 (12)	4	—
Periconceptional only	11	1 (9)	10	2 (20)
Unknown/Missing	5	—	2	—
**Maternal symptom status**				
Asymptomatic	366	37 (10)	56	7 (13)
Symptomatic	176	23 (13)	25	11 (44)
Unknown	4	—	—	—
**Trimester of pregnancy loss**				
Pregnancy loss, first trimester	NA	NA	28	10 (36)
Pregnancy loss, second trimester	NA	NA	35	3 (9)
Pregnancy loss, third trimester	NA	NA	17	5 (29)
Missing	NA	NA	1	—
**Completed pregnancies reported to the U.S. Zika Pregnancy Registry**^¶¶^ **(n = 449)**				
**Characteristic**	**Live births (n = 414)**		**Pregnancy losses (n = 35)**	
**Total**	**414**	**60 (14)**	**35**	**18 (51)**
Possible Zika virus–associated birth defects***				
Birth defects reported	30	16 (53)	4	2 (50)
No birth defects reported	384	44 (11)	31	16 (52)

**Abbreviations:** IgM = immunoglobulin M; NA = not applicable; PRNT = plaque-reduction neutralization test; RT-PCR = reverse transcription–polymerase chain reaction.

* Includes placental specimens (placenta, fetal membranes, or umbilical cord) for all 546 live births and infant autopsy specimens for six of nine neonatal deaths. For pregnancy losses (spontaneous abortions, terminations, and stillbirths), includes placental specimens (placenta, fetal membranes, or umbilical cord) for 62 and fetal specimens for 58 pregnancy losses; both fetal and placental tissues were submitted for 38 cases.

**^†^**Tissue RT-PCR positive = at least one placental or fetal tissue specimen was positive by Zika virus RT-PCR.

^§^ Confirmed recent Zika virus infection = positive Zika virus RT-PCR, or Zika or dengue virus IgM positive or equivocal with Zika virus plaque-reduction neutralization test (PRNT) titer ≥10 and dengue virus PRNT titer <10; Recent unspecified flavivirus infection = negative or no Zika virus RT-PCR performed, with Zika or dengue virus IgM positive, or equivocal with Zika virus and dengue virus PRNT titers ≥10; Maternal samples negative by Zika virus IgM, all or part of possible exposure occurred >12 weeks before serum collection date = negative or no Zika virus RT-PCR performed; Zika virus IgM negative with all or part of possible exposure occurring >12 weeks before serum collection date; Pending/Unknown = Test results unknown or pending; No evidence of Zika virus infection = Zika or dengue virus IgM positive or equivocal with Zika virus PRNT titer <10 regardless of dengue virus PRNT titer, or Zika IgM negative where all possible exposure occurred within 2–12 weeks of serum collection date. Applies to results of testing on maternal clinical specimens (e.g., serum, urine). Only includes results of Zika virus clinical laboratory testing conducted in the United States and U.S. territories.

^¶^ Includes nine live births with negative maternal Zika virus IgM and Zika and dengue virus PRNT titers ≥10.

** Includes two live births with negative maternal Zika virus RT-PCR on serum or urine where all or part of possible exposure occurred >12 weeks before specimen collection date and no Zika virus IgM testing was performed.

^††^ Confirmed congenital Zika virus infection = positive Zika virus RT-PCR, Zika virus IgM positive and Zika virus PRNT titer ≥10; Probable congenital Zika virus infection = Zika virus IgM-positive, no PRNT titers reported, or Zika and dengue virus PRNT titers ≥10; Negative infant Zika virus test results = neither Zika virus RT-PCR nor Zika virus IgM positive results; No infant specimen test results reported = testing could be not performed, not reported, or pending. Applies to results of testing on infant or fetal clinical specimens (e.g., serum, cord blood, urine, cerebrospinal fluid, amniotic fluid), however if infant PRNT titers not available, maternal serum PRNT titers were used. Only includes results of Zika virus clinical laboratory testing conducted in the United States and U.S. territories.

^§§^ Trimester of infection or possible exposure is based on symptom onset date for symptomatic pregnant women, and for asymptomatic women was based on trimester(s) of suspected vectorborne or sexual exposure. Periconceptional exposure only is defined as infection or possible exposure during the 8 weeks before conception (6 weeks before and 2 weeks after the first day of the last menstrual period).

^¶¶^ U.S. Zika Pregnancy Registry inclusion criteria = Pregnant women with laboratory evidence of Zika virus infection (positive or equivocal test results, regardless of whether they have had symptoms) and periconceptionally, prenatally, or perinatally exposed infants born to these women, and infants with laboratory evidence of congenital Zika virus infection (positive or equivocal test results, regardless of whether they had symptoms) and their mothers (https://www.cdc.gov/zika/reporting/registry.html).

*** Birth defects include those that met the U.S. Zika Pregnancy Registry surveillance case definition for birth defects potentially associated with Zika virus infection during pregnancy as of May 18, 2017. These birth defects include brain abnormalities and/or microcephaly, intracranial calcifications, ventriculomegaly, neural tube defects and other early brain malformations, eye abnormalities, or other consequences of central nervous system dysfunction including arthrogryposis (joint contractures), clubfoot, congenital hip dysplasia, and congenital deafness (https://www.cdc.gov/zika/geo/pregnancy-outcomes.html).

**FIGURE:**
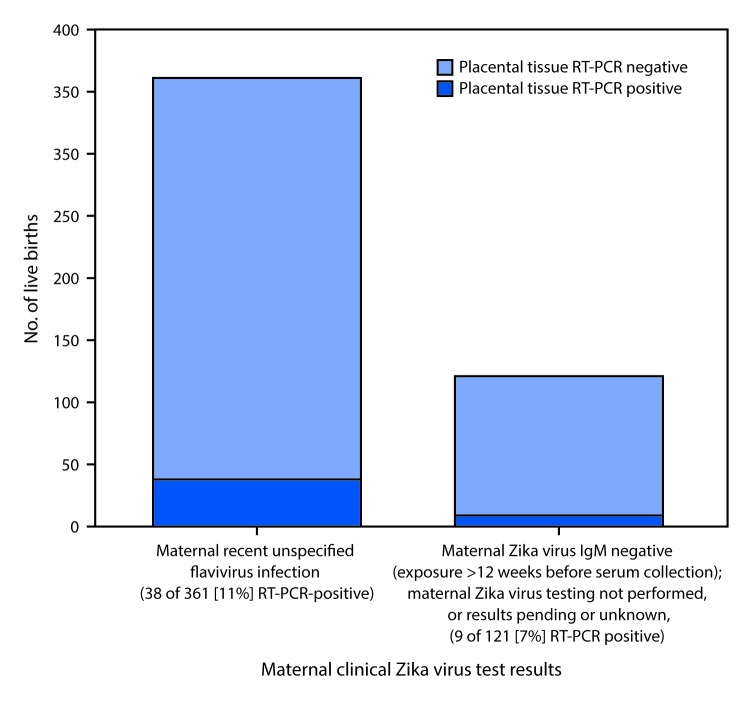
Zika virus placental tissue RT-PCR results, among live births with neither clinical laboratory evidence of confirmed recent Zika virus infection on maternal testing nor confirmed congenital Zika virus infection on infant testing (n = 482),*^,†,§^ by maternal clinical Zika virus test results categories^¶^^,^** — 50 U.S. states and the District of Columbia, January–December, 2016 **Abbreviations:** IgM = immunoglobulin M; PRNT= plaque-reduction neutralization test; RT-PCR = reverse transcription–polymerase chain reaction. * Excludes live births with confirmed recent maternal Zika virus infection (positive Zika virus RT-PCR, or Zika or dengue virus IgM-positive or equivocal with Zika virus PRNT titer ≥10 and dengue virus PRNT titer <10) or no evidence of Zika virus infection (Zika or dengue virus IgM positive or equivocal with Zika virus PRNT titer <10 regardless of dengue PRNT titer, or Zika virus IgM negative where all possible exposure occurred within 2–12 weeks of serum collection date), or confirmed congenital Zika virus infection based on infant testing (positive Zika virus RT-PCR or Zika virus IgM positive and Zika virus PRNT titer ≥10 with dengue virus PRNT titer <10). ^†^ Includes 41 live births where infants had laboratory evidence of probable congenital Zika virus infection; 9/41 (22%) with placental tissue RT-PCR positive; and 441 live births where infants had negative Zika virus testing or no Zika virus testing reported; 38/441 (9%) with placental tissue RT-PCR positive. Positive placental tissue RT-PCR results provide evidence of confirmed recent maternal Zika virus infection. ^§^ Placental tissue RT-PCR positive = at least one placental tissue specimen was positive by Zika virus RT-PCR. ^¶^ Recent unspecified flavivirus infection = negative or no Zika virus RT-PCR performed, with Zika or dengue virus IgM positive, or equivocal with Zika and dengue virus PRNT titers ≥10. ** Maternal samples negative by Zika virus IgM, all or part of possible exposure occurred >12 weeks before serum collection date with negative or no Zika virus RT-PCR performed, maternal Zika virus testing not performed, or results pending or unknown.

Placental or fetal tissues from 18 (22%) of the 81 pregnancy losses tested positive for Zika virus by RT-PCR, including 4/13 (31%) with recent unspecified maternal flavivirus infection, 2/18 (11%) with negative maternal Zika virus IgM, where possible maternal exposure occurred >12 weeks before serum collection, and 1/16 (6%) with no maternal clinical samples tested (Table 2). Among 14 pregnancy losses with no evidence of maternal Zika virus infection, no placental or fetal tissues tested RT-PCR–positive. Ten of 28 (36%) first trimester pregnancy losses and 5/17 (29%) third trimester pregnancy losses were tissue RT-PCR–positive, compared with only 3/35 (9%) second trimester losses (Table 2). However, 13/28 (46%) first trimester pregnancy losses had evidence of confirmed recent maternal Zika virus infection from clinical specimens, compared with 5/35 (14%) of second trimester and 1/17 (6%) third trimester pregnancy losses.

Among the 627 completed pregnancies included in this report, 449 (72%) were included in the USZPR (Table 2). Thirty live births were reported to have possible Zika virus–associated birth defects. Sixteen of these (53%) were Zika virus RT-PCR–positive on placental tissues; however, a positive placental tissue RT-PCR cannot distinguish between maternal and congenital infection. Ten of these 16 had recent unspecified maternal flavivirus infection, and six had negative maternal Zika virus IgM, where possible maternal exposure occurred >12 weeks before serum collection. Among nine live births with negative maternal Zika IgM, where possible maternal exposure occurred >12 weeks before serum collection, and placental tissue RT-PCR was positive, six had possible Zika virus–associated birth defects.

## Discussion

Among live births, placental tissue RT-PCR provided confirmation of recent maternal Zika virus infection for 47 (10%) women who otherwise did not have a definitive diagnosis. Given the complexity of Zika virus testing and interpretation, tissue specimen analysis provides another opportunity to confirm maternal Zika virus infection. A definitive maternal diagnosis of Zika virus infection provides valuable information to guide the evaluation and management of infants with possible congenital exposure.

Placental tissue RT-PCR testing was positive in a relatively low proportion of live births with recent unspecified maternal flavivirus infection (10%) or negative maternal Zika virus IgM on serum collected >12 weeks after possible exposure (10%). Placental testing might provide additional diagnostic information and can continue to be considered in these scenarios (https://www.cdc.gov/zika/pdfs/placental-testing-guidance.pdf), depending on the availability of public health resources. The yield of Zika virus testing of placental tissues should continue to be reassessed as additional data are collected.

Placental tissues have both maternal and fetal components, and Zika RT-PCR cannot discriminate between viral RNA from maternal and fetal areas (*9*). Although placental testing cannot confirm or exclude congenital Zika virus infection, infants might be more likely to receive appropriate clinical evaluation when a mother has confirmed recent Zika virus infection. Negative placental RT-PCR results do not rule out maternal or congenital Zika virus infection; evaluation of pregnant women and infants for Zika virus in accordance with CDC guidance is essential to direct appropriate infant clinical management and follow-up (*3*,*4*). Infant Zika virus testing and neuroimaging should not be delayed while results of placental testing are pending.

Among live births with possible Zika virus–associated birth defects reported to the USZPR and included in this analysis, 53% were Zika virus RT-PCR–positive on placental tissues. The implications of a positive placental Zika virus RT-PCR for infant clinical outcomes are currently unknown. However, further study could explore the relationship between the presence of Zika virus RNA in placental specimens, fetal infection, and development of possible Zika virus–associated birth defects.

In this report, Zika virus IHC was only positive on fetal and placental tissues from first and second trimester pregnancy losses. Zika virus IHC-positivity in brain tissues from infant deaths has been reported in other studies (*9*,*10*). Although all IHC-positive cases were also RT-PCR–positive, IHC can provide valuable insight into viral localization and pathogenesis in pregnancy losses and infant deaths.

The findings in this report are subject to at least five limitations. First, a negative Zika virus RT-PCR on placental tissues does not exclude maternal Zika virus infection. Factors that could lead to false-negative results include levels of viral RNA below the limit of assay detection, variability in tissue sampling, and degradation of viral RNA because of insufficient tissue fixation or prolonged formalin-fixation.^††^ Second, pregnancy outcomes in this analysis might not be representative of all pregnancies with possible Zika virus exposure, maternal Zika virus infection, or Zika virus–associated birth defects in the United States. Pregnancies ending in a loss or with fetuses or infants with birth defects might be more likely to have tissue specimens submitted, particularly among pregnancies with negative maternal Zika virus IgM >12 weeks after possible exposure. Third, possible testing bias limits the ability to compare placental test results by results of infant clinical laboratory testing, because infants with possible Zika virus–associated birth defects might be more likely to have Zika virus testing performed. Fourth, the approach to testing of placental and fetal tissues changed over time, which might have resulted in variability in testing bias over the reporting period. Changes included routinely testing tissue specimens for completed pregnancies where maternal Zika virus IgM was negative >12 weeks after possible exposure (beginning in August 2016) (*3*,*4*), and focusing testing of placental specimens from live births on those without a confirmed recent maternal Zika virus infection diagnosis (https://www.cdc.gov/zika/pdfs/placental-testing-guidance.pdf). Finally, clinical, epidemiologic, and laboratory information reflects data reported to USZPR and CDC’s IDPB as of the date of this report, and might be incomplete.

These findings describe the contributions of testing placental and fetal tissue specimens for Zika virus infection to the diagnosis of maternal infection. Although the proportion of live births with placental tissues positive for Zika virus by RT-PCR was low, tissue analysis can be valuable when maternal serologic testing either cannot differentiate between Zika virus and other related flaviviruses, or has been conducted >12 weeks after possible maternal exposure, and infant Zika virus testing is not definitive, negative, or not performed. Tissue analysis provides another opportunity to confirm maternal Zika virus infection, which can be important to both families and health care providers. However, because a positive Zika virus RT-PCR on placental tissues cannot distinguish between maternal and congenital infection, following current CDC guidance for clinical diagnostic testing and management of pregnant women with possible Zika virus exposure and infants with possible congenital Zika virus infection continues to be important (*3*,*4*).

**Summary**

**What is already known about this topic?**

Zika virus infection during pregnancy can cause microcephaly and other brain abnormalities. Diagnosis of Zika virus infection is challenging because of serologic cross-reactivity with other related flaviviruses and limited duration of viremia. Zika virus RNA can be detected in placental and fetal tissues, which can provide an opportunity to diagnose maternal Zika virus infection and can be considered when maternal serologic testing is not definitive or is negative outside the optimal testing window.

**What is added by this report?**

In the 50 U.S. states and District of Columbia, placental testing provided a confirmed diagnosis of recent maternal Zika virus infection for 10% of live births with possible maternal exposure to Zika virus that lacked definitive evidence of a maternal or congenital Zika virus infection. This included pregnancies with clinical laboratory evidence of recent unspecified maternal flavivirus infection, and those with negative maternal Zika virus IgM, where possible maternal exposure occurred >12 weeks before serum collection.

**What are the implications for public health practice?**

Testing of placental tissues from live births provided definitive evidence of maternal Zika virus infection. Although the proportion of live births for which placental tissue was RT-PCR–positive for Zika virus was relatively low, testing of placental tissues from live births can continue to be considered when results of maternal Zika virus testing are not definitive or testing is not performed within the optimal time. Ensuring appropriate Zika virus testing and clinical follow-up of infants, according to published CDC guidance is critical in order to identify congenital Zika virus infection.

## Footnotes

* Possible exposure to Zika virus includes: 1) travel to or residence in an area at risk for Zika virus transmission and with a CDC travel notice, or 2) condomless sexual exposure to a partner who traveled to or lived in an area with risk of Zika virus transmission and a CDC travel notice during pregnancy or the periconceptional period (https://www.cdc.gov/zika/geo/index.html).

† Infant laboratory evidence categories apply to results of testing on infant or fetal clinical specimens (e.g., serum, cord blood, urine, cerebrospinal fluid, amniotic fluid), however if infant PRNT titers were not available, maternal serum PRNT titers were used. Categories include the following: confirmed congenital Zika virus infection = positive by Zika virus RT-PCR, Zika virus IgM positive and Zika virus PRNT titer ≥10; probable congenital Zika virus infection = Zika virus IgM-positive, no PRNT titers reported, or Zika and dengue virus PRNT titers ≥10; negative infant Zika virus test results = neither Zika virus RT-PCR nor Zika virus IgM positive results; no infant specimen test results reported = testing could be not performed, not reported, or pending. Only includes results of Zika virus clinical laboratory testing conducted in the United States and U.S. territories (https://wwwn.cdc.gov/nndss/conditions/zika/case-definition/2016/06/).

§ Trimester of infection or possible exposure is based on symptom onset date for symptomatic pregnant women or trimester(s) of suspected vectorborne or sexual exposure for asymptomatic pregnant women. Periconceptional exposure only is defined as infection or possible exposure during the 8 weeks before conception (6 weeks before and 2 weeks after the first day of the last menstrual period).

¶ U.S. Zika Pregnancy Registry inclusion criteria = pregnant women with laboratory evidence of Zika virus infection (positive or equivocal test results, regardless of whether they have had symptoms) and periconceptionally, prenatally, or perinatally exposed infants born to these women, and infants with laboratory evidence of congenital Zika virus infection (positive or equivocal test results, regardless of whether they have symptoms) and their mothers (https://www.cdc.gov/zika/reporting/registry.html).

** Birth defects include those that met the USZPR surveillance case definition for birth defects potentially associated with Zika virus infection during pregnancy as of May 18, 2017. These birth defects include brain abnormalities and/or microcephaly; intracranial calcifications; ventriculomegaly; neural tube defects and other early brain malformations; eye abnormalities; or other consequences of central nervous system dysfunction including arthrogryposis (joint contractures), clubfoot, congenital hip dysplasia, and congenital deafness (https://www.cdc.gov/zika/geo/pregnancy-outcomes.html).

†† Recommendations for specimen collection and submission are available at https://www.cdc.gov/zika/laboratories/test-specimens-tissues.html.
